# When Meaning Is Not Enough: Distributional and Semantic Cues to Word Categorization in Child Directed Speech

**DOI:** 10.3389/fpsyg.2017.01242

**Published:** 2017-07-19

**Authors:** Sara Feijoo, Carmen Muñoz, Anna Amadó, Elisabet Serrat

**Affiliations:** ^1^Department of Modern Languages and English Studies, University of Barcelona Barcelona, Spain; ^2^Department of Psychology, University of Girona Girona, Spain

**Keywords:** semantic cues, distributional cues, word categorization, child-directed speech, grammatical categories

## Abstract

One of the most important tasks in first language development is assigning words to their grammatical category. The *Semantic Bootstrapping Hypothesis* postulates that, in order to accomplish this task, children are guided by a neat correspondence between semantic and grammatical categories, since nouns typically refer to objects and verbs to actions. It is this correspondence that guides children’s initial word categorization. Other approaches, on the other hand, suggest that children might make use of distributional cues and word contexts to accomplish the word categorization task. According to such approaches, the *Semantic Bootstrapping* assumption offers an important limitation, as it might not be true that all the nouns that children hear refer to specific objects or people. In order to explore that, we carried out two studies based on analyses of children’s linguistic input. We analyzed child-directed speech addressed to four children under the age of 2;6, taken from the CHILDES database. The corpora were selected from the Manchester corpus. The corpora from the four selected children contained a total of 10,681 word types and 364,196 word tokens. In our first study, discriminant analyses were performed using semantic cues alone. The results show that many of the nouns found in parents’ speech do not relate to specific objects and that semantic information alone might not be sufficient for successful word categorization. Given that there must be an additional source of information which, alongside with semantics, might assist young learners in word categorization, our second study explores the availability of both distributional and semantic cues in child-directed speech. Our results confirm that this combination might yield better results for word categorization. These results are in line with theories that suggest the need for an integration of multiple cues from different sources in language development.

## Introduction

One of the most significant challenges for children when learning their first language is assigning words to their corresponding syntactic categories. For instance, how do English-learning children know that ‘table’ is a noun, ‘eat’ is a verb, and ‘kiss’ can be both a noun and a verb? Generativist approaches have put forward the so-called *Semantic Bootstrapping Hypothesis* (Pinker, 1984; Fodor, 1998; Laurence and Margolis, 2001), which predicts that children use semantic information to map words into their corresponding grammatical category. In particular, children are said to have innately specified information in terms of nouns referring to objects, and verbs referring to actions. The present paper undertakes a critical examination of these assumptions: on the one hand, it tests the reliability of semantic information by examining the amount of nouns in children’s input that refer to specific objects or people; on the other hand, it examines the accuracy with which words could be categorized on the basis of a combination of multiple cues (i.e., both semantic and distributional cues).

The strength of the semantic bootstrapping approach lies on the idea that mappings between semantics and grammatical classes are universal (e.g., nouns denote objects while verbs denote actions in any language). On the contrary, other types of cues like phonological or distributional cues are language-specific. In particular, the noun-object mapping seems easily attainable in language development, as many studies have highlighted a noun bias in children’s early vocabularies across different languages, mainly because of the conceptual simplicity that nouns exhibit (Jackson-Maldonado et al., 1993; Bates et al., 1994; Caselli et al., 1999; Bassano, 2000; Gleitman et al., 2005). Concreteness or imageability might be the underlying predictor of the identifiability of nouns from their observed extralinguistic contexts. Thus, learners’ noun bias might be based on the assumption that object-reference items are the best ones that fit in a word-to-world pairing procedure. With the meaning of nouns, and the intuition that nouns relate to real-world objects, children might then start building a rudimentary nominal grammatical category.

Then, the semantic bootstrapping proposal claims that one source of information about the meaning of words is available from the beginning of the language learning process, and it constitutes the basis from which learners start building their initial grammatical categories. This initial information source allows the learner to acquire a subset of lexical items (i.e., nouns that refer to specific objects) which requires little linguistic knowledge and is pragmatically supported. Thus, the grounding of grammatical categories would start from the identification of semantic categories first, and semantic features would later on bootstrap grammar.

Nevertheless, the main problem within the semantic bootstrapping approach is that it presupposes that correlations between semantics and grammatical categories are perfect mappings in any language. Furthermore, such an approach also assumes that children start the language learning process with the expectation that such mappings actually exist. However, this assumed straightforward concept-word pairing is somehow problematic, as some have pointed out (Ambridge and Lieven, 2011).

To start with, naive learners with no prior knowledge of grammatical categories in their language and who are exposed to fluent speech might even fail to perform the object referent-noun mapping. Even if child learners were ready to map external referents to particular words in the input, how does the child know which word does the “object” semantic component refer to, out of the possible words she hears? First of all, the words to which children are exposed might refer to objects which are absent from the child’s sight when they are spoken (Gleitman, 1990). In addition, when addressing children, parents may refer to the same object with different words in different contexts (Yurovsky et al., 2012). Furthermore, the mapping task becomes even less clear when facing multiple-word utterances (Yu and Ballard, 2007). However, such utterances are the ones which children are most likely to encounter in the course of their linguistic development, as only a small percentage of utterances in child-directed speech have been reported to contain words in isolation (Bernstein Ratner and Rooney, 2001; Monaghan and Christiansen, 2010; Monaghan and Mattock, 2012; Feijoo and Hilferty, 2013). Furthermore, research shows that mothers never or hardly ever use words in isolation, even in situations where they are explicitly teaching new vocabulary to their children (Aslin et al., 1996).

A further problem concerns the way the object-word mapping itself should proceed. It is known as *Quine’s Gavagai problem*, or the problem of referential indeterminacy (Quine, 1960). Imagine a mother-child interaction situation where the mother would point to a brown running dog and say “*Look at the dog*!”. Even if the visual image and the target word *dog* were immediately associated, how does the child know that the word *dog* actually refers to the dog itself and not, say, to a more general referent such as *animal*, or to a specific type of dog, or to a part of the dog (i.e., its legs, its tail…), or to a physical property of the dog (i.e., its colour, its fur…), or to the action of running itself? How do children know that they can equally use the word *dog* to refer to another type of dog (i.e., a sitting dog, a white dog, a dog from a different breed, etc.) and they cannot use it with other animals like a brown running cat? A mere world-to-word mapping assumption cannot account for children’s choice and learning of the word *dog* and its natural referents in the real world.

Recent empirical evidence has shown that cross-situational statistical learning might be the key to solve the referential ambiguity problem illustrated in the *Gavagai problem* (Smith and Yu, 2008; Scott and Fisher, 2012; Vlach and Johnson, 2013; Suanda et al., 2014; Benitez et al., 2016). This statistical learning approach suggests that adults as well as young children are able to map linguistic units to referents in the world by tracking co-occurrence probabilities across different learning situations. Thus, it seems that learners are sensitive to the statistical consistency with which a given word is used in front of a given referent and the mapping between heard word and seen referent can occur in this way.

However, the noun-object mapping proposal also assumes that the set of nouns which children hear from their input specifically refer to objects in the real world. What if children were exposed to superordinate terms? Or what if they were exposed to abstract words whose meaning does not relate to a specific object or referent? Traditional linguistic analyses postulate that the category “noun” is both a notional and a grammatical concept (Lyons, 1977). There is a central semantic concept of noun, which is present in all languages, and which includes words for persons, animals, and things. All the other more abstract ontological categories denoted by nouns appear to be generalizations from this core concept.

Acknowledging that this core concept includes only a subset of all possible nouns gives rise to the question of how large the proportion of nouns belonging to this subset actually is in English. In other words, can this subset account for all the examples of nouns that English-learning children are exposed to? Previous studies have already pointed out that semantic criteria alone do not provide a reliable basis to determine the category membership of many words in English, since there are many nouns which do not denote physical objects (e.g., *an explanation*) (Yu and Ballard, 2007; Tare et al., 2008), or there are many words in English which can be both classified as nouns or verbs (e.g., *a kiss* vs. *to kiss*, *a walk* vs. *to walk*, etc.) (Nelson, 1995; Maratsos, 1999; Tomasello, 2010; Conwell and Morgan, 2012).

In this line, Nelson et al. (1993) propose two distinguishable semantic classes of nouns: on the one hand, BLOCS neatly correspond to basic level object categories; on the other hand, XBLOCS refer to all those words which would naturally fall out of the cognitive basic level, either because their extralinguistic referent is too general or because they do not refer to a specific object referent at all. Nelson et al. (1993: p. 71) further distinguished different types of XBLOCS taking their meaning into account:

• *Locations*: places, both indoors and out (e.g., *beach*, *kitchen*)
• *Actions*: single actions, specific or general (e.g., *kiss*, *help*)
• *Superordinate/generic*: terms that denote what are generally considered superordinate categories (e.g., *toys*, *animals*).
• *Events*: terms that refer to complex events that take place through time (e.g., *lunch*, *party*).
• *Person roles*: roles that people play in social/cultural life (e.g., *doctor*, *brother*).
• *Natural phenomena*: states, actions, events or entities (e.g., *sky*, *snow*, *clouds*).
• *Temporal entities*: e.g., *morning*, *day*.
• *Quantities*: e.g., *drop*.

In their study, Nelson et al. (1993) showed that children learn and use many XBLOC words early in the language learning process. As multi-word combinations and productivity in noun morphology develops, noun roles are assigned to these words, showing that they are accurately categorized as nouns. Therefore, words which lack the expected semantic content that would yield their successful categorization (and which would therefore be left unclassified on the basis of semantics alone) are nonetheless accurately classified in their right word class from the early stages of the nominal category building process.

Other studies also point out that object nouns or action verbs do not necessarily dominate children’s earliest lexical productions, neither in English, nor in other languages (Gopnik and Choi, 1995; Bassano, 2000). And still, those words which do not neatly map into their corresponding semantic category are correctly classified by children and they are used in a grammatical way.

Given the evidence provided, it seems clear that there are other types of cues at work, alongside semantics, when words are being classified into their corresponding grammatical category. For instance, at least as far as English is concerned, several studies have provided evidence for the usefulness of phonological information as a key element for the access to grammatical properties of the language (e.g., Kelly, 1996; Monaghan et al., 2005, 2007; Fitneva et al., 2009). These findings suggest that, on the one hand, these phonological cues are reliably found in parents’ language. On the other hand, evidence has also been found that young language learners as well as adults are aware of such cues and their correlation to grammatical categories.

Besides, syntactic or distributional information might be a very powerful cue that assists young language learners in language development as well. Regarding word categorization, the context of a word with respect to other words in the same sentence might provide indications about the category of that word in English. For example, English nouns are typically preceded by determiners and followed by nominal morphology (e.g., ***the*** *babies*), while verbs are typically preceded by auxiliaries or strong subject pronouns and followed by verbal morphology (e.g., ***she*** *walked*).

Studies on computer simulations have provided evidence for the usefulness of distributional and positional information for an initial categorization of words in the absence of semantic or referential information (Cartwright and Brent, 1997; Redington et al., 1998). Such distributional information appears to be available not only to adult speakers but also to young language learners as well (Mintz, 2003; Monaghan et al., 2007; Feijoo et al., 2015).

Furthermore, empirical evidence from artificial language studies seems to suggest that children’s learning of grammatical structure as well as their word-reference associations improve when words are coherently marked by a combination of different types of cues, either phonological, distributional or semantic cues (Gomez and Lakusta, 2004; Gerken et al., 2005; Lany and Gomez, 2008; Lany, 2014). However, when words are not reliably marked by these cues, infants fail to learn their semantic or grammatical properties, since young language learners are more likely to learn from deterministic rather than probabilistic cues, and they would only rely on relatively robust correlations between word-forms and their corresponding grammatical category (Lany and Saffran, 2010; Yurovsky et al., 2012; Lany, 2014).

In particular, Lany and Saffran (2010) found that experience with reliable distributional cues in the input is a key factor that predicts children’s learning of word meanings: when words’ distributional properties correctly indicated the grammatical category to which words belonged, infants successfully learned word-referent mappings. In contrast, infants failed at the word-referent pairing task when distributional cues were not reliably correlated with grammatical category membership.

The main goal of the present study was to test the likelihood with which children could classify all the nouns they hear in their corresponding grammatical category using semantic cues derived from the input. To this end, two studies were carried out: in the first one, a corpus-based analysis of child-directed speech explores the potential strength of semantic information alone in children’s input. The second study examines the benefits that a combination of semantic and distributional information could provide to language learning children when facing the task of word categorization.

## Study 1

### Objective

While it is not still clear whether the first analysis that children perform on the input is on notional grounds (and therefore, semantic cues are considered first) or distributional grounds (and thus, syntactic cues are considered first), it is widely accepted that the semantic notion of *object* and *action* might assist language learners in the identification of nouns and verbs, respectively. As mentioned earlier, one of the problems for the *Semantic Bootstrapping* proposal (Pinker, 1984) lies on the difficulty of identifying the meaning of unknown words and, consequently, their semantic category. Besides, the links between semantic categories and grammatical categories are not one-to-one but many-to-many (Mintz, 2003). Thus, for example, not all items within the semantic category of actions are verbs. An adjective such as *noisy*, or a noun such as *call*, can also be semantically classified as actions. In fact, as Nelson et al. (1993) have pointed out, the actual proportion of English nouns which conform to the semantic category of objects is only a small subset of the whole noun inventory.

Acknowledging that the core traditional definition of nouns as labels for people, animals, and things only includes a subset of all English nouns raises the question of what is the actual percentage of nouns which belong to this subset (i.e., how big the subset is, considering all English nouns). It also raises the issue of whether this smaller subset can be taken to account for all of the nouns that young children are exposed to and will later acquire.

Thus, the main objective of this first study is to examine the usefulness and reliability of semantic cues for the categorization of nouns in English child-directed speech. While it is true that not all English nouns refer to objects exclusively, it is also true that the kind of interactions that very young children are involved in are often restricted to the *here* and *now* and to familiar objects within each child’s reach (Baldwin, 1993; Clark, 2009). A close evaluation to words which semantically refer to objects will be performed in order to test how large the set of object-referring nouns actually is in children’s input. Furthermore, words which semantically refer to actions will also be analyzed in order to examine the possible overlap between the grammatical categories of nouns and verbs which have semantic content in common.

### Corpus Preparation

We analyzed child-directed speech addressed to four children under the age of 2;6, taken from the CHILDES database (MacWhinney, 2000). The corpora were selected from the Manchester corpus (Theakston et al., 2001) and included the files from Aran, Carl, Anne, and Becky. The characteristics of the selected corpora are summarized in **Table 1**.

**Table 1:** Characteristics of the selected corpora.

	Anne	Aran	Becky	Carl
Age range	1;10.07 to 2;6.29	1;11.12 to 2;6.17	2;0.07 to 2;6.29	1;8.22 to 2;6.19
Sex	Female	Male	Female	Male
Total types	2,761	3,432	2,222	2,266
Total tokens	103,457	118,469	65,411	76,859

All the lexical items from each corpus were then classified into two different categories to be analyzed separately. One category included all nouns, and the other category, the “non-noun” category, included all verbs, adjectives, and adverbs. For dual-class words, that is, English words that can, for instance, be both classified as nouns and verbs (e.g., *kiss*, *call*, *brush*), the KWAL utility of the CLAN program was used in order to work out the exact number of tokens that were used as nouns and the number of tokens that were used as verbs in every transcript. **Table 2** shows a summary of the characteristics of each group, with the total number of types and the total number of tokens found in each group. It also shows the Type/Token ratio as an indicator of lexical diversity. However, note that such indicator will not be a variable considered in our analyses.

**Table 2:** Characteristics of the groups of lexical items from all the corpora.

	Total types	Total tokens	TTR
Total all corpora	10,681	364,196	0.029
Total selected lexical items	9,621	139,624	0.069
Total nouns	5,397	51,577	0.104
Total non-nouns	4,233	88,047	0.048

### Cue Derivation

All the lexical items from the child corpora were further classified into different groups, according to the semantic features they bore. The main goal was to analyze the consistency and reliability with which the relationship between semantic information and grammatical categories is represented in the input addressed to English-learning children. Particular attention was paid to the semantic overlap between nominal elements which describe actions and prototypical verbal elements which equally describe actions. Such contradictory information might be especially misleading for any child who relies on semantic information to form grammatical categories, since they would wrongly classify action nouns as verbs.

A set of semantic cues which have been said to identify nouns (Nelson et al., 1993) was selected. Not only the group of nouns, but also the set of verbs, adjectives, and adverbs from the “non-noun” group in the selected corpora, were tested against the selected cues. This made it possible to analyze the degree of overlap between semantic and grammatical categories and, therefore, to work out the risk of misclassifying elements into their wrong grammatical category on the basis of semantic information. Following the work by Nelson et al. (1993), the set of semantic cues that were selected for the analysis include the following:

• *Sem1*: this was the group for proper nouns, which semantically refer to single individuals and never expand to whole-class reference. A number of classical studies (e.g., Katz et al., 1974; Gelman and Taylor, 1984) have already provided evidence for the fact that children understand proper nouns and common nouns differently from very early stages of language development. Proper nouns of people, animals, toys, stories, songs, places, holidays, etc., scored 1 in this category and 0 everywhere else.
• *Sem2*: this was the category that best corresponded with the traditional notional definition of nouns as labels for names of people, animals, and objects. In particular, the *Sem2* group was meant to include all common count nouns that could be considered members of the basic level category in Rosch’s (1978) terms (e.g., *dog*, *apple*, *chair*). Words from the corpora which matched this definition scored 1 here and they scored 0 everywhere else.
• *Sem3*: this was the category that included all mass nouns in the corpora (e.g., *milk*, *paper*). They differ from the nouns from the *Sem2* category in the sense that *Sem3* words do not denote discrete entities but whole substances. This difference is also reflected in syntax, since mass nouns do not combine with the same type of determiner as count nouns. Furthermore, children seem to be aware of such differences by their second year of life (Soja, 1992). Words that matched the *Sem3* description scored 1 here and they scored 0 everywhere else.
• *Sem4*: this was the category which Nelson et al. (1993) labeled as XBLOCS (i.e., not basic level object categories). These were words which could not be included in the *Sem3* category or in the *Sem2* category. While Nelson et al. (1993) distinguish several different types of XBLOC nouns, for the purpose of the present study only two different groups of the *Sem4* category were made:

- *Sem4a*: this was the group for words that described actions (e.g., *kiss*, *help*, *trip*). Nouns that matched this description scored 1 here and 0 in the other *Sem* categories. Crucially, verbs which matched this description also scored 1 here.
- *Sem4b*: this group included all the other XBLOC nouns from the study by Nelson et al. (1993). Among them were words which denoted locations and places, both indoors and outdoors (e.g., *kitchen*, *park*, *school*); words which described generic categories and belonged to the superordinate level in Rosch’s (1978) terms (e.g., *animal*, *thing*); words which referred to abstract events or social gatherings (e.g., *lunch*, *party*); words which described person roles (e.g., *doctor*, *brother*); words which denoted natural phenomena (e.g., *sky*, *heat*); words which referred to temporal entities (e.g., *morning*, *day*); and words which designated quantities (e.g., *drop*, *spoonful*). Then, words that semantically referred to any of these groups scored 1 in this category and 0 in all the other *Sem* categories.

What motivates the division of the *Sem4* group into two subgroups is that, unlike Nelson et al. (1993), the purpose of our analysis is not to provide an accurate description and classification of XBLOC nouns in general. Instead, the main objective is to test the amount of English nouns that lack a direct semantic component available to language learners (i.e., which nouns are XBLOC nouns and which ones are not). Besides, it was also important to analyze the degree of semantic overlapping between nouns and other words such as verbs, for which the *Sem4a* category was created.

In order to guarantee reliability on coding, words were classified into their corresponding semantic categories by two different raters. Inter-rater reliability was measured using the Cohen’s Kappa statistic (*Kappa* = 0.926, *p* < 0.05).

### Results and Discussion

The set of nouns and the set of non-nouns which were previously obtained were tested using the four different semantic cues described above. The total number of nouns that met each of the semantic descriptions under consideration is shown in **Table 3**. In a similar way, **Table 4** shows the results obtained from the equivalent analysis with the rest of open class words. As shown in the table, while most non-nouns (i.e., a total of 2,520) belong to the *Sem4a* group, since they denote actions, the remaining 1,654 non-nominal types were not captured by any of the semantic features under consideration.

**Table 3:** Total number of nouns in each semantic category.

	Total types	Total tokens	TTR
Proper nouns (*Sem1*)	699	9,516	0.073
Basic level count nouns (*Sem2*)	2,675	25,369	0.105
Basic level mass nouns (*Sem3*)	288	2,655	0.108
Action words (*Sem4a*)	228	1,419	0.161
Non-basic level words (*Sem4b*)	1,507	12,618	0.119

**Table 4:** Total number of non-nouns in each semantic category.

	Total types	Total tokens	TTR
Proper nouns (*Sem1*)	0	0	–
Basic level count nouns (*Sem2*)	0	0	–
Basic level mass nouns (*Sem3*)	0	0	–
Action words (*Sem4a*)	2,520	46,530	0.054
Non-basic level words (*Sem4b*)	59	1,318	0.045

Correct classification of all types and tokens with all the semantic cues was tested with discriminant analyses. A discriminant analysis is a multivariate inferential technique. Its main objective is to classify individuals in two groups according to a number of previously selected variables. It works out the reliability with which the variables accurately describe the members of a given group and whether presence or absence of a given set of variables determines group membership. Regarding types, when the variables *Sem1*, *Sem2*, *Sem3*, and *Sem4a* were entered simultaneously, overall correct classification reached 82.0%, Wilks λ = 0.439, χ^2^ = 7916.359, *p* < 0.001. However, this high overall correct classification was obtained mainly because of the high score obtained in the correct classification of non-noun words, which was 98.7%. This indicates that there were almost no verbs, adjectives or adverbs which carried any of the semantic features which are typically associated to nouns. However, correct classification among nouns lowered to 67.9%.

The same analysis that was run with types was also run with all tokens from the four corpora. For the token analysis, when the same semantic cues were entered simultaneously, only 39.9% of nouns were correctly classified. Overall correct classification was 66.3% of tokens, Wilks λ = 0.945, χ^2^ = 544.696, *p* < 0.001. As with types, overall correct classification was relatively high because of the high scores in the non-noun group, with identical results in the token analysis as in the type analysis. However, such high scores in the non-noun group only indicate that the group of nouns which could be potentially created on the basis of semantic information is very accurate (i.e., there are very few non-noun words which are at risk of being misclassified as nouns on the basis of their semantic content). On the contrary, completeness scores (i.e., the number of nouns which are correctly classified as such) are relatively low, which indicates that only a subset of nouns would be correctly classified as nouns on the basis of their meaning, and most nouns would be wrongly classified as non-nouns.

These analyses show that there is an important number of noun tokens which lack the semantic features with which nouns are associated, and whose semantic information is either ambiguous or too broad. Thus, children would not be able to work out the grammatical category to which these nouns belong on the basis of semantic information alone. On the contrary, other sources of information might be necessary for the correct categorization of most of the nouns to which children are exposed. Our second study tested the likelihood with which semantic and syntactic cues together could yield better results than semantic cues alone for word categorization.

## Study 2

### Objective

As seen from the results of Study 1, the semantic notion of “object” only correlates with the grammatical category of nouns in a very weak way. The purpose of this second study is, then, to find a second source of information which, alongside with semantic information, might assist young English learners in the categorization of the nouns they hear from the input. In this line, Yu (2006) found that the association between words and objects was assisted by the presence of syntactic information. Furthermore, analyses of child-directed speech corpora highlight the usefulness of multiple cue integration for word categorization (Monaghan et al., 2007; Monaghan and Mattock, 2012; Yurovsky et al., 2012). In more general terms, several studies also highlight the importance of redundant information in language learning (e.g., Gogate et al., 2000; Frank et al., 2009; Smith et al., 2010; Riordan and Jones, 2011). Thus, combined cues seem to provide better language learning outcomes.

As mentioned earlier in the introduction, recent findings from artificial language experiments (Lany and Saffran, 2010; Lany, 2014) also suggest that the presence of robust correlations between the distributional and semantic properties of words enhances infants’ word learning and word categorization. Thus, the objective of this second study was to test how robust these correlations between semantic and distributional cues actually are in natural child-directed speech, given that it significantly differs from artificial languages in terms of complexity and the presence of “noisy” elements.

### Corpus Preparation

For the second study, we used the same corpora as for Study 1. We also followed the same procedure and criteria regarding the classification of lexical items into the “noun” and the “non-noun” groups.

### Cue Derivation

The same semantic cues that were used in Study 1 were also used in Study 2. Regarding distributional cues, following previous studies (Mintz, 2003; Monaghan et al., 2007; Feijoo et al., 2015), for the present analysis we only considered the set of syntactic contexts which included extremely local grammatical relationships of the type *Determiner* + *Noun* (i.e., English articles, demonstrative determiners, possessive determiners, and quantifiers preceding nouns).

A list of six different distributional contexts was generated and every target word was analyzed to see whether its context matched any of the six established. Words scored 1 if they appeared in any of those syntactic contexts and they scored 0 otherwise. In this sense, this analysis with distributional cues was slightly different from the one using semantic cues. In terms of semantic cues, when a word (type or token) scored 1 in a given category, it scored 0 in all other categories as well (e.g., a word cannot be a proper noun (category *Sem1*) and a common object (category *Sem2*) at the same time). The same is true of the Token analysis using distributional cues. However, as far as the Type analysis using distributional cues is concerned, it is not true that when a Type scores 1 in a given distributional context it scores 0 in all the other contexts as well. For instance, we found occurrences of the phrase *a dog* and the phrase *the dog* in the corpus of the same child. In terms of tokens, both instantiations of the word *dog* correspond to two different tokens, with their respective different syntactic context each. However, in terms of types, the same word type *dog* is both found in two different syntactic contexts. That is why, as a Type, *dog* scored 1 in both contexts at the same time (for further examples see the Supplementary Material, with an excerpt of the classified material).

In order to obtain the different distributional contexts in which every word in our corpus occurred, the COOCCUR utility of the CLAN program was used to generate a list of every word in our corpus plus the word which occurred immediately before it, as well as the overall token frequency of every obtained pair. The set of distributional contexts considered include the following:

• *Syn0*: this grouped together words which were not preceded by any element which prototypically introduces nouns in English (e.g., articles, possessive determiners, etc.). Thus, this category included nouns which occurred with no distributional context (or at least not a context that would assist word categorization) as well as verbs, adjectives, and adverbs. Words in the corpora whose distributional context matched this description scored 1 here and 0 in all the other contexts.
• *Syn1*: this grouped together words preceded by an indefinite article (e.g., ***a*** *dog*, ***an*** *apple*). Words within such a context scored 1 here and 0 elsewhere. Since it is local syntactic contexts that are being considered, an adjective such as *beautiful* in *a beautiful girl* would also score 1 here.
• *Syn2*: this was meant to group together words which were introduced by a definite article (e.g., ***the*** *toys*). Words found in such a syntactic context scored 1 here and 0 elsewhere.
• *Syn3*: words preceded by a demonstrative determiner were classified in this group (e.g., ***this*** *man*, ***those*** *birds*, etc.). Words within such a distributional context scored 1 here and 0 elsewhere.
• *Syn4*: this category included all words in the corpora which were preceded by any form of possessive determiner (i.e., ***my*** *hand*, ***your*** *teddy*, etc.). Words within such distributional contexts scored 1 in this category and 0 in all other categories.
• *Syn5*: this category was meant to group items which were introduced by any English quantifier (e.g., ***many*** *things*, ***some*** *cookies*, etc.). Items found within such contexts scored 1 in this category and 0 everywhere else.

### Results and Discussion

Parallel to the analysis with semantic cues in Study 1, for the analysis of distributional cues, the set of nouns and the set of non-nouns obtained from the corpora were tested using the distributional contexts described above. The total number of nouns that were found in each of the distributional contexts established is shown in **Table 5**, while **Table 6** shows the results obtained from the equivalent analysis with the rest of open class words.

**Table 5:** Total of nouns in each of the distributional contexts.

	Total types	Total tokens	TTR
**bbb + x** (Syn0)	4,311	23,651	0.357
**{a, an} + x** (Syn1)	1,459	6,414	0.227
**{the} + x** (Syn2)	2,194	10,815	0.254
**{DEMONSTR.} + x** (Syn3)	895	2,674	0.377
**{POSSESSIVE} + x** (Syn4)	1,120	4,554	0.248
**{QUANTIFIER} + x** (Syn5)	1,221	3,473	0.357

**Table 6:** Total of non-nouns in each of the distributional contexts.

	Total types	Total tokens	TTR
**bbb + x** (Syn0)	4,380	81,300	0.131
**{a, an} + x** (Syn1)	346	2,009	0.172
**{the} + x** (Syn2)	228	1,112	0.205
**{DEMONSTR.} + x** (Syn3)	433	1,615	0.268
**{POSSESSIVE} + x** (Syn4)	142	329	0.432
**{QUANTIFIER} + x** (Syn5)	528	1,682	0.314

As in Study 1, the categorization potential of the selected variables was assessed by means of a discriminant analysis. In order to examine the effects of the interaction between distributional and semantic cues, the set of six distributional variables and the four semantic variables considered in this second study were introduced together as predictor variables in the discriminant function. Regarding types, when the combination of cues was introduced, there were 90.1% of correctly classified noun types and 96.8% of correctly classified non-noun types. Overall correct classification was 93.0% of all types, Wilks λ = 0.312, χ^2^ = 11196.554, *p* < 0.001.

The same classificatory system made up of the combination of distributional and semantic variables was tested with the set of tokens from the four child corpora. When all the variables were introduced simultaneously as predictor variables in a standard discriminant analysis, there were 47.9% of correctly classified noun tokens and 98.3% of correctly classified other open class word tokens. Overall correct classification reached 70.1%, Wilks λ = 0.899, χ^2^ = 1028.161, *p* < 0.001.

As can be seen, the results obtained with a combination of semantic and distributional cues are higher than the ones obtained with semantic cues only, both with types and tokens. Furthermore, higher scores of correct noun classification as well as correct non-noun classification are also obtained with the combination of distributional and semantic cues than with distributional cues alone (Feijoo et al., 2015).

In terms of accuracy (i.e., number of non-nouns which are correctly classified as such), the high results obtained in Study 1 are replicated here and they are not affected by the presence of distributional variables. In this way, we can claim that, on the basis of the information available in the input and the way grammatical categories are represented, children are very unlikely to misclassify verbs, adjectives or adverbs in the noun category. This would make a noun category very accurate and with a very low chance of including non-noun elements.

In terms of completeness, the results obtained in Study 2 indicate that children are more likely to create a more complete nominal category (i.e., one that includes many more nominal elements) when using distributional and semantic cues at the same time, rather than when using semantic cues alone. The analysis with types when using cues in combination reveals a very high proportion of correctly classified nouns (and, therefore, a very low risk of there being a misclassification of noun types in the non-noun group).

The analysis with tokens provides correct noun classification scores which are higher when using cues in combination rather than when using semantic cues alone. Even if such correct classification scores with noun tokens are still slightly below 50%, evidence from previous studies (Marchman and Bates, 1994; Bybee, 1995; Maratsos, 2000) suggests that regular morphosyntactic patterns are generalized once patterns exhibit a relative type frequency. Thus, children appear to be more attentive to type frequency than to token frequency, and they are more likely to generalize from distributional contexts that appear on many stems than those that appear on only a few stems, even when the token instantiations of those fewer stems have an overall higher frequency. High token frequency is useful to keep an irregular form, but does not make a paradigm productive. On the other hand, type frequency helps language learners to identify productive paradigms (Clark, 2009; Ambridge and Lieven, 2011). All in all, we could claim that distributional and semantic cues available in child-directed speech interact in such a productive way as to allow the classification of most nouns in their correct grammatical category.

## General Discussion

As seen earlier, the objective of the first study described in the present paper was to analyze the potential usefulness of semantic information as far as the categorization of English nouns is concerned. Traditional accounts on noun categorization based on semantic information have put forward the idea that young language learners might group all nouns together under the semantic label of “object” and all verbs together under the semantic label of “action” (Pinker, 1984). The fact that most nouns refer to common objects and their subsequent imageability based on notional grounds has been argued to be the reason why nouns are learned before verbs or before words encoding actions and relations in language development (Caselli et al., 1999; Bassano, 2000; Gleitman et al., 2005).

However, more recent findings show that nominal elements denoting common objects dominate children’s early vocabularies only as far as types are concerned (Nelson et al., 1993; Gopnik and Choi, 1995; Nelson, 1995). However, reports on early vocabulary production show that there are more tokens of non-nominal expressions (i.e., verbs and relational words such as *there*, *up* or *no*) than of nouns, and that this tendency is not only true of the English language (Gopnik and Choi, 1995).

Thus, as the authors suggest, the makeup of children’s early lexicons might not be the result of there being a more “learnable” or more “imaginable” category in semantic terms, but it might be a reflection of children’s actual linguistic experience, in such a way that children’s first words might be instantiations of the kind of words that their parents used with them. This is also coherent with the results obtained from the input analysis undertaken in the present studies. As seen earlier (see **Table 2**), the descriptive data that was obtained from the corpus preparation reveal that the kind of linguistic experience to which the four children under consideration are exposed contains far more nominal types than non-nominal types (i.e., there were overall 5,388 noun types and 4,233 other open class word types). However, when it comes to tokens, other open class words exceed nominal tokens by far (i.e., the four corpora together contained 51,577 nominal tokens, but 88,047 other open class word tokens).

The predominance of noun types over other open class word types has been explained by the fact that many very complex and abstract entities are realized as nouns in adult language (Nelson, 1995). This raises the question of how many of these nominal types are actually “easily learnable.” If children engage in word categorization tasks guided by the fact that all words that refer to common objects are grouped together under the noun category, then how many of the overall 5,388 noun types considered in the present study can actually be described by these semantic features and how salient is that proportion in statistical terms?

Previous analyses of linguistic input addressed to young language learners have shown that common object nouns represent only a very small proportion of all the noun repertory that children hear (Nelson et al., 1993; Nelson, 1995; Monaghan and Mattock, 2012). The initial prediction as far as the first study was concerned was that many of the nouns to which very young English-learning children are exposed refer to basic-level common objects, and will be subsumed by variable *Sem2*. Other nouns were expected to either refer to proper names of people (i.e., *Sem1* nouns) or to non-discrete mass entities (i.e., *Sem3* nouns). Neither of them would pose any learning problems either. However, an important number of nominal elements were also predicted to lack any of the above-mentioned semantic characteristics (i.e., *Sem4* nouns). Without the necessary semantic components, those nouns would not guide children in their categorization tasks, provided children perform such tasks on the basis of semantic information.

These predictions are confirmed by the data obtained in the first analysis. The descriptive data from the first study show that only approximately half of the noun types as well as half of the nominal tokens refer to basic-level object nouns and were described by variable *Sem2*, while the other half belonged to the other three semantic subsets. Within those, about a third of the noun types and a quarter of the noun tokens were included in variable *Sem4*, which was the one which grouped together all nouns which did not have any of the semantic features that would foster correct grammatical categorization on the basis of semantic information alone. The results obtained from the discriminant analyses performed with types as well as with tokens confirm this weak correlation between semantic information and grammatical category assignment as far as nouns are concerned. Thus, for the type analysis, only 67.9% of the nominal types were correctly classified, and these completeness scores dropped dramatically in the token analysis, with only 39.9% of correctly classified nominal tokens.

A further objective in this first study was to see whether there was an overlap between nouns and other open class words as far as semantic information is concerned, and to test whether the overlap was significant enough so as to bring about a considerable misclassification of elements. Provided that, according to Pinker (1984), children engage in a semantic analysis of the input and make the hypothesis that all words that denote actions belong to the grammatical category of verbs, what do children do when they encounter action words which are not verbs? And what do they do with verbs that do not denote actions? Does the input offer a high proportion of contradictory information of this kind?

The present analysis shows that, indeed, there is a slight degree of semantic overlap between nouns and other open class words, since some of the XBLOC nouns have certain semantic features which are typical of verbs (i.e., mainly nouns that denote actions). At the same time, obviously not all of the other open class words denoted actions since, besides non-action verbs, adjectives, and adverbs were also included in this group. Thus, in the case of semantic information, the kind of overlap between nouns and other open class words seems to be one way, that is, some of the nouns might lack the corresponding nominal semantic features, or might have verbal semantic features, and might therefore be misclassified as other open class words (i.e., mainly misclassified as verbs). However, the same risk does not seem to hold for any of the other open class words, since they are very unlikely to contain any of the semantic features associated to nouns, and thus be misclassified as such on the basis of semantic information. Empirical analyses of children’s early vocabularies also suggest that, even when the very same word can be both a noun and a verb (e.g., English *kiss*, *hug*, *call*, *help*) most children assign those action words exclusively to the verb category, regardless of their parents’ frequent use of them as nouns (Nelson, 1995). As mentioned earlier, the discriminant analyses using semantic variables that were performed on all types and tokens confirm this, since the accuracy scores that were obtained in all cases were very close to 100% of correctly classified other open class words.

The fact that correct classification scores were far better with types than with tokens confirms the tendency described above in connection to early vocabulary production, where higher productivity of nominal types is observed, while tokens from other categories outnumber nominal tokens. Thus, children’s early word production can be seen as a direct reflection of the kind of linguistic environment that they have experienced. However, nouns cannot be claimed to be “easily learnable” on the basis of their semantic association to basic-level objects alone, since statistical analyses where the diagnosticity of such semantic classification was tested provided a considerable proportion of misclassified nouns.

These findings suggest that semantic information may not always be the only factor which is used to determine the assignment of words to their grammatical category. Furthermore, as other studies have also suggested (Monaghan et al., 2007; Monaghan and Mattock, 2012; Yurovsky et al., 2012), learners never hear speech with just a single kind of cue to word categorization in isolation. In children’s natural linguistic environment, there are multiple redundant language-specific cues to word category membership.

When distributional and semantic cues were put to interact with one another in the second study, overall successful categorization scores were expected to improve when compared to the results obtained with semantic cues in isolation. This prediction was born out by the results obtained from the second study. The interaction between semantic and distributional cues gave more successful results than semantic cues in isolation. The results obtained regarding successful categorization with both kinds of cues in combination were also higher than the results obtained in previous studies using distributional cues alone (Mintz, 2003; Monaghan et al., 2007; Feijoo et al., 2015). In this sense, the results from both our first and our second study suggest that semantic cues contributed in providing an accurate grammatical category of nouns, since very few other open class words are at risk of being misclassified as nouns. On the other hand, distributional cues might have contributed in providing greater completeness scores, with a larger number of nouns being correctly classified as nouns, since the low completeness scores obtained from semantic cues alone were improved.

Monaghan et al. (2007) have already proposed the *Phonological-Distributional Coherence Hypothesis* in their analysis with distributional and phonological cues. According to them, both sources of information contribute differently toward word classification. Other studies have also highlighted the benefits of multiple types of cue for word categorization as well as for other language learning tasks (Monaghan et al., 2007; Smith et al., 2010; Riordan and Jones, 2011; Monaghan and Mattock, 2012; Yurovsky et al., 2012). Thus, having a combined number of different variables seems to increase the likelihood with which a given element will be successfully encoded and learned by children.

On the other hand, having to attend to several types of cues does not seem to imply an increase in terms of difficulty or cognitive processing demand on the part of very young language learners, at least as far as the combination of semantic and distributional information is concerned. The empirical evidence available to date seems to suggest that children are able to attend to multiple cues and use them for language learning tasks from a very early age (Thiessen and Saffran, 2003). In particular, by 14 months, infants have been described to be able to use determiners to identify nouns (Höhle et al., 2004; Shi and Melançon, 2010). Furthermore, hearing a word in a predictable distributional context promotes its identification for adults as well as for young infants (Lany, 2014). Thus, young language learners may also more readily encode a novel noun when it is preceded by a determiner. Facility with encoding would also make it easier for infants to determine the referent of the novel noun, and to form a robust mapping between the noun and the object it refers to, provided such mapping exists.

Therefore, on the basis of the evidence provided by the data obtained in our studies, we can claim that the combination of semantic and distributional information found in natural child-directed speech could significantly contribute to the correct categorization of most of the nouns to which English-learning infants are exposed. However, the results reported in our two studies are restricted to the kind of English speakers considered in our analyses. There might be important differences -both in terms of quantity and quality- in the input used with infants exposed to languages other than English, or infants from cultures other than western cultures and who are conventionally spoken to in a different way. Thus, for example, other languages with less distributional regularities might exploit phonological or prosodic information that might assist young language learners in their word categorization tasks. Further research on child-directed speech by non-English speakers should shed some light on how the results obtained from the present studies would generalize cross-linguistically and cross-culturally.

## Author Contributions

SF, CM, AA, and ES: Contributions to the conception and design; analysis and interpretation of data; drafting the work and revising it critically; final approval of the version to be published; agreement to be accountable for all aspects of the work in ensuring that questions related to its accuracy or integrity are appropriately resolved.

## Conflict of Interest Statement

The authors declare that the research was conducted in the absence of any commercial or financial relationships that could be construed as a potential conflict of interest.
