# Supplementary material for: When Meaning Is Not Enough: Distributional and Semantic Cues to Word Categorization in Child Directed Speech
Source: Front Psychol. 2017 Jul 19;8:1242. doi: 10.3389/fpsyg.2017.01242 (PMC5516671; doi:10.3389/fpsyg.2017.01242)
Supplement: Supplementary file 1 [file Data_Sheet_1.DOCX]

**Appendix**: an excerpt of the classified material based on the two categorizations.

| **Token** |  |  |  |  |  |  |  |  |  |  |  |  |
| --- | --- | --- | --- | --- | --- | --- | --- | --- | --- | --- | --- | --- |
| **Freq.** | **Word** | **Sem1** | **Sem2** | **Sem3** | **Sem4a** | **Sem4b** | **Syn0** | **Syn1** | **Syn2** | **Syn3** | **Syn4** | **Syn5** |
| 4 | accident |  |  |  |  | 4 | 1 | 3 |  |  |  |  |
| 10 | air |  |  |  |  | 10 | 1 |  | 9 |  |  |  |
| 1 | airport |  |  |  |  | 1 |  |  | 1 |  |  |  |
| 1 | alexander | 1 |  |  |  |  | 1 |  |  |  |  |  |
| 1 | alf_thomson | 1 |  |  |  |  | 1 |  |  |  |  |  |
| 26 | amy | 26 |  |  |  |  | 26 |  |  |  |  |  |
| 2 | angel |  |  |  |  | 2 |  |  |  |  |  | 2 |
| 15 | animal |  |  |  |  | 15 | 7 | 3 | 1 | 2 |  | 2 |
| 34 | animals |  |  |  |  | 34 | 8 |  | 18 | 4 |  | 4 |
| 13 | anna | 13 |  |  |  |  | 13 |  |  |  |  |  |
| 1 | annabel | 1 |  |  |  |  | 1 |  |  |  |  |  |
| 6 | annie | 6 |  |  |  |  | 6 |  |  |  |  |  |
| 30 | apple |  | 30 |  |  |  | 5 | 16 | 3 | 2 | 2 | 2 |
| 7 | apples |  | 7 |  |  |  | 6 |  |  |  |  | 1 |
| 7 | arm |  | 7 |  |  |  | 1 |  |  |  | 5 | 1 |
| 1 | armie |  | 1 |  |  |  |  |  |  |  |  | 1 |
| 2 | armies |  | 2 |  |  |  | 1 |  |  |  | 1 |  |
| 9 | arms |  | 9 |  |  |  |  |  |  |  | 9 |  |
| 1 | attention |  |  |  |  | 1 |  |  |  |  |  | 1 |
| 1 | aubergine |  | 1 |  |  |  |  | 1 |  |  |  |  |
| 1 | aunty_carol | 1 |  |  |  |  | 1 |  |  |  |  |  |
| 1 | aunty_pam | 1 |  |  |  |  | 1 |  |  |  |  |  |
| 3 | aunty_shirley | 3 |  |  |  |  | 3 |  |  |  |  |  |
| 39 | bag |  | 39 |  |  |  | 10 | 5 | 13 | 6 | 3 | 2 |
| 4 | bags |  | 4 |  |  |  | 1 |  | 1 |  |  | 2 |
| 29 | ball |  | 29 |  |  |  | 16 | 4 | 3 |  | 1 | 5 |
| 21 | balloon |  | 21 |  |  |  | 5 | 3 | 7 | 3 | 3 |  |
| 4 | balloons |  | 4 |  |  |  |  |  | 4 |  |  |  |
| 1 | balls |  | 1 |  |  |  |  |  |  |  |  | 1 |
| 39 | banana |  | 39 |  |  |  | 7 | 16 | 1 | 6 | 6 | 3 |
| 7 | bananas |  | 7 |  |  |  | 5 |  |  |  |  | 2 |
| 16 | basket |  | 16 |  |  |  | 1 |  | 15 |  |  |  |
| 1 | baskets |  | 1 |  |  |  | 1 |  |  |  |  |  |
| 1 | bat |  | 1 |  |  |  |  | 1 |  |  |  |  |
| 1 | battery |  | 1 |  |  |  |  |  |  |  |  | 1 |
| 1 | batterys |  | 1 |  |  |  |  |  |  |  |  | 1 |
| 1 | beach |  |  |  |  | 1 |  |  | 1 |  |  |  |
| 9 | beans |  | 9 |  |  |  | 4 |  |  |  |  | 5 |
| 21 | bear |  | 21 |  |  |  | 13 | 5 | 2 |  |  | 1 |
| 35 | bed |  | 35 |  |  |  | 17 | 8 | 2 |  | 8 |  |
| 6 | bed+room |  |  |  |  | 6 |  |  | 1 |  | 4 | 1 |
| 1 | bee |  | 1 |  |  |  |  |  |  |  |  | 1 |
| 2 | bees |  | 2 |  |  |  |  |  | 2 |  |  |  |
| 1 | beginning |  |  |  |  | 1 |  |  | 1 |  |  |  |
| 12 | ben | 12 |  |  |  |  | 12 |  |  |  |  |  |
| 22 | bert | 22 |  |  |  |  | 22 |  |  |  |  |  |
| 6 | bertie | 6 |  |  |  |  | 6 |  |  |  |  |  |
| 14 | bike |  | 14 |  |  |  | 2 | 4 | 1 |  | 7 |  |
| 4 | bill | 4 |  |  |  |  | 4 |  |  |  |  |  |
| 1 | billy | 1 |  |  |  |  | 1 |  |  |  |  |  |
